# Long COVID in pediatric age: an observational, prospective, longitudinal, multicenter study in Italy

**DOI:** 10.3389/fimmu.2025.1466201

**Published:** 2025-04-09

**Authors:** Susanna Esposito, Matteo Puntoni, Michela Deolmi, Greta Ramundo, Giuseppe Maglietta, Marco Poeta, Stefania Zampogna, Claudia Colomba, Agnese Suppiej, Fabio Cardinale, Samantha Bosis, Elio Castagnola, Fabio Midulla, Carlo Giaquinto, Paola Giordano, Giacomo Biasucci, Valentina Fainardi, Francesco Nunziata, Roberto Grandinetti, Anna Condemi, Giuseppe Raiola, Alfredo Guarino, Caterina Caminiti, Long-Covid-Ped Italian Study Group

**Affiliations:** ^1^ Pediatric Clinic, University Hospital, Department of Medicine and Surgery, University of Parma, Parma, Italy; ^2^ Clinical and Epidemiological Research Unit, University Hospital of Parma, Parma, Italy; ^3^ Pediatric Infectious Disease Unit, Department of Maternal and Childealth, University Hospital "Federico II", Naples, Italy; ^4^ Department Pediatrics, Hospital of Crotone, Crotone, Italy; ^5^ Division of Pediatric Infectious Diseases, "G. Di Cristina" Hospital, ARNAS Civico Di Cristina Benfratelli, University of Palermo, Palermo, Italy; ^6^ Pediatric Clinic, University of Ferrara, Ferrara, Italy; ^7^ Complex Operating Unit Paediatrics, Giovanni XXIII Paediatric Hospital, University of Bari, Bari, Italy; ^8^ S.C. Pediatria-Pneumoinfettivologia, Fondazione IRCCS Ca' Granda Ospedale Maggiore Policlinico, Milan, Italy; ^9^ Pediatric Infectious Diseases Unit, IRCCS Istituto Giannina Gaslini, Genoa, Italy; ^10^ Department of Maternal, Infantile and Urological Sciences, Sapienza University of Rome, Rome, Italy; ^11^ Division of Pediatric Infectious Diseases, Department of Women's and Children's Health, University of Padua, Padua, Italy; ^12^ Department of Interdisciplinary Medicine, Pediatric Section, "Aldo Moro" University of Bari, Bari, Italy; ^13^ Pediatrics and Neonatology Unit, Department of Medicine and Surgery, University of Parma, Guglielmo da Saliceto Hospital, Piacenza, Italy; ^14^ Department of Pediatrics, "Pugliese-Ciaccio" Hospital, Catanzaro, Italy

**Keywords:** long COVID, pediatric infectious diseases, post-COVID symptoms, SARS-CoV-2, neurological dysfunction

## Abstract

**Introduction:**

This observational prospective longitudinal multicenter study examines the occurrence and characteristics of long COVID (LC) in the Italian pediatric population.

**Methods:**

Conducted across 12 Pediatric Units in Italy from January to March 2022, the study involved 1129 children diagnosed with SARS-CoV-2 infection. Data were collected via self-administered questionnaires at 1-3 months, 3-6 months, and 6-12 months post-infection, with LC defined as symptoms persisting for at least 2 months and occurring 3 months post-diagnosis.

**Results:**

Results revealed that 68.6% of children reported at least one post-COVID symptom, with 16.2% experiencing LC. The most frequent symptoms included respiratory issues (43.4%), neurological and cognitive dysfunction (27.7%), gastrointestinal symptoms (22.1%), fatigue (21.6%), and sleep disturbances (18.8%). Age and gender differences were significant, with older children and females more prone to cardiovascular and neurological & cognitive dysfunction.

**Discussion:**

The study highlights that LC in children presents similarly to adults, though less frequently. The occurrence of LC was lower compared to adult populations, likely due to the generally milder course of COVID-19 in children. The findings underscore the need for targeted follow-up and support for affected children, especially considering the long-term persistence of symptoms. Further research is necessary to explore the impact of COVID-19 vaccines on pediatric LC and the effects of different SARS-CoV-2 variants. These insights are crucial for developing strategies to manage and mitigate long-term impacts in children recovering from COVID-19.

## 1 Introduction

The persistence or development of signs and symptoms of disease in patients with previous SARS-CoV-2 infection weeks or months after the end of the acute phase of COVID-19 has been reported since the early stages of the pandemic (1–3). To identify adult patients with long COVID (LC) and facilitate studies aimed at defining the clinical and prognostic characteristics of this condition, several definitions of LC have been developed (4, 5). These definitions were generally based on the clinical characteristics of COVID-19 in adult subjects. Unfortunately, the definitions of LC varied significantly in the number and type of symptoms included, as well as the duration of symptoms (7). Despite most studies adopting the World Health Organization (WHO) definition (8), comparing the results of these studies remained difficult. It was established, however, that LC was very common in adults, with up to 50% of patients with previous SARS-CoV-2 infection, even if asymptomatic, potentially suffering from LC (5, 9). The clinical presentation of LC could significantly vary, with more than 200 signs and symptoms involving different organs and body systems reported with varying degrees of severity (7, 8). Common symptoms included fatigue, malaise, altered smell and taste, breathlessness, and cognitive impairments (10). Moreover, it was evidenced that, especially in the most severe and prolonged cases, LC could lead to significant socioeconomic problems for patients and their families, mainly due to severe work difficulties (11).

Studies in children were initially few, mainly because pediatric COVID-19 was considered a very mild disease generally evolving without significant complications (12–14). SARS-CoV-2 infection in children is frequently asymptomatic, and symptomatic cases are generally mild, presenting as an uncomplicated upper respiratory tract infection that resolves spontaneously in a few days (15). Hospitalization rates in children are significantly lower than in adults, and pediatric deaths constitute only 0.4% of all COVID-19 deaths worldwide (16). Despite later evidence that children can develop a late-onset, sometimes very severe disease such as the multisystem inflammatory syndrome (MIS-C), post-infectious manifestations of COVID-19 remained poorly studied for several months (17, 18). Furthermore, the evaluation of the results of the available studies was complicated by the heterogeneity of the studies themselves (19).

To identify children with LC, criteria prepared for adults were initially used, complicating the pooling and analysis of results. The frequency and clinical manifestations of LC in children remained undefined. As COVID-19 differs in children compared to adults, it was considered highly likely that a specific definition of pediatric LC should be used to identify affected children (20, 21). To address this problem, Seylanova et al. used a three-phase online Delphi process followed by an online consensus meeting to develop a definition for LC in children (22). The Seylanova criteria were developed to address the limitations of the WHO definition for long COVID in children and adolescents (8), which was largely based on adult data and lacked specificity for the pediatric population. The aim of this descriptive prospective, longitudinal, multicenter study is to evaluate long COVID occurrence in the Italian pediatric population and assess the characteristics and persistence of specific symptoms according to the criteria of Seylanova et al. (22).

## 2 Materials and methods

### 2.1 Study design

Thisdescriptive, prospective, longitudinal, multicenter study involved 12 Pediatric Units in Italy. Enrollment was conducted from January 1, 2022, to March 31, 2022 in the coordinating center, and subsequently in the participating centers, throughout 2022. Patient follow-up occurred at intervals of 1-3 months, 3-6 months, and 6-12 months post-diagnosis of SARS-CoV-2 infection. This study was conducted in accordance with the Declaration of Helsinki and was initially approved by the Ethics Committee of Area Vasta Emilia Nord (approval number 952/2021/OSS/AOUPR, dated December 15, 2021), followed by approval from the Ethics Committees of all participating centers. Informed written consent was obtained from both parents (or legal guardians) of the enrolled children, and from patients aged 6 years and older.

### 2.2 Study population

Each participating pediatric unit enrolled patients with confirmed SARS-CoV-2 infection, including those diagnosed and managed in both outpatient and inpatient settings. Patients under 18 years of age were enrolled within three months of a confirmed SARS-CoV-2 infection diagnosis, verified by reverse transcription polymerase chain reaction (RT-PCR). Both hospitalized patients and those managed on an outpatient basis at the participating centers were included. During the enrollment visit, data were collected after obtaining signed informed consent forms through a self-administered questionnaire designed for pediatric and adolescent populations by the International Severe Acute Respiratory and Emerging Infection Consortium (ISARIC), based on a pre-existing adult questionnaire.

Data collection was organized into two phases. Initially, families were asked to complete the Level 1 survey, which gathered basic demographic information (sex, ethnicity, height, weight, number of family members, parents' ages, occupation, education level), clinical history, comorbidities, signs and symptoms of the acute phase of SARS-CoV-2 infection, treatments received, patient and caregiver-reported outcomes, hospitalization rates, vaccination status, quality of life, and the development of specific (respiratory failure, shock, myocarditis, acute renal failure, coagulopathy, multiple organ failure, pediatric multisystem inflammatory syndrome, intussusception, diabetic ketoacidosis) and non-specific clinical manifestations (asthenia, sleep quality, personal experiences related to COVID-19). **Supplementary Table 1** shows details on the categorisation of post-COVID-19 symptoms.

In cases where patients reported persistent symptoms such as fever, cough, dyspnea, asthenia, emotional distress, abnormal behaviors, or cognitive disorders, additional data were collected through personalized, standardized questions (Level 2 survey).

Follow-up data were collected at three intervals post-acute infection: 1-3 months, 3-6 months, and 6-12 months. Participants and their caregivers completed follow-up questionnaires online via a link, on paper forms during medical visits or sent by mail, or through telephone interviews. Long COVID was defined as symptoms persisting for at least 2 months, occurring 3 months post-diagnosis, and not explained by another diagnosis.

### 2.3 Statistical analysis

Demographic and clinical characteristics of enrolled patients were analyzed using descriptive statistics. Outcomes reported by patients and/or caregivers were categorized and analyzed by gender and age cohorts at diagnosis (0-5 years, 6-11 years, 12-17 years). For continuous variables, the mean and standard deviation (SD) or median and interquartile range (IQR) were calculated; for categorical variables, frequencies and percentages were determined. Differences in proportions between patient groups were tested using the chi-square test or Fisher's exact test. The outcomes were also measured adopting time-to-event analyses, considering as event the first symptom recorded in the observation period of any subject. The cumulative symptom-free survival was thus estimated with the Kaplan–Meier method, considering the time from the first survey to the first symptom recorded; censoring was applied to the last available follow-up time point (last survey compiled) in the absence of symptoms. Log-rank test was used to test for differences among age classes (test for trend) or between males and females (simple test). Median follow-up time was calculated by adopting the reverse Kaplan–Meier method. A p-value of less than 0.05 was considered statistically significant. All statistical analyses were performed centrally using STATA (StataCorp. 2023. Stata Statistical Software: Release 18. College Station, TX: StataCorp LLC.).

## 3 Results

### 3.1 Demographics

The study included 1,129 children diagnosed with SARS-CoV-2 infection who completed the first questionnaire (1–3 months after the first COVID-19 symptom), of whom 854 (75.6%) completed the second (3–6 months), and 729 (64.6%) completed the third (6–12 months).

Subjects’ characteristics are shown in **Table 1**. Of the 1,129 children who completed the first questionnaire, 124 (11%) required hospitalization for COVID-19. The average age of the participants was 7.7 years, with an age range from 0 to 17 years. The gender distribution was nearly equal, with 52% males and 48% females. The age groups were categorized as follows: 0-1 years (12%), 2-5 years (17%), 6-11 years (54%), and 12-17 years (18%). Among the participants at the initial survey, 241 children (21.4%) had received at least one dose of the COVID-19 vaccine, with an average of 1.91 doses per vaccinated individual.

**Table 1:** Subjects’ characteristics (n=1,129).

Characteristic	N. (%)
Male sex, n (%)	586 (51.9)
Age, years, mean (SD)	7.7 (4.4)
Age classes	
0-1	137 (12.1)
2-5	186 (16.5)
6-11	604 (53.5)
12-17	202 (17.9)
COVID-19 vaccination status (at least 1 dose), n (%)	241 (21.4)
<ns/> doses, mean (SD)	1.92 (0.6)
At least 1 comorbidity prior to COVID-19 diagnosis	416 (36.9)
Main (>1%) types of comorbidities, n (%)	
Atopic dermatitis/Eczema	132 (11.7)
Allergic rhinitis/hay fever	125 (11.1)
Asthma	51 (4.5)
Bowel/stomach problems	42 (3.7)
Food allergies	42 (3.7)
Respiratory diseases (excluding asthma)	36 (3.2)
Neurological diseases/Neurodisabilities	32 (2.8)
Endocrine diseases	25 (2.2)
Heart disease	16 (1.4)
Genetic conditions	15 (1.3)
Sickle cell anemia	12 (1.1)
Born prematurely (<37 weeks)	103 (9.1)
Receiving mental health support	53 (4.7)

SD, standard deviation.

Overall, 416 patients (36.9%) had comorbidities. The most frequently reported comorbidities included atopic dermatitis (132 patients, 11.7%), rhinitis (125 patients, 11.1%), asthma (51 patients, 4.5%), gastrointestinal manifestations (42 patients, 3.7%), and food allergies (42 patients, 3.7%). Additionally, 103 patients (9.1%) were born prematurely, and 53 patients (4.7%) were receiving mental health support prior to the pandemic.

### 3.2 Occurrence of post-COVID symptoms

**Table 2** summarizes the distribution of post-COVID symptoms and long COVID symptoms by category. Overall, 68.6% of the children reported at least one post-COVID-19 symptom, with 16.2% experiencing long COVID. Respiratory symptoms were the most frequently reported, with 43.4% of participants affected and 7.4% having symptoms lasting over three months. Neurological and cognitive dysfunction was the second most common issue, reported by 27.7% of participants, with 4.5% experiencing symptoms for more than three months. Gastrointestinal symptoms were reported by 22.1% of participants, with 3.2% lasting over three months. Other common symptoms included fatigue (21.6%, with 3.5% lasting over three months), sleep disturbances (18.8%, with 2.7% lasting over three months), and poor appetite (15.4%, with 2.0% lasting over three months)​​.

**Table 2:** Number of subjects with post-COVID-19 symptoms, by category, n (%).

Symptoms	All symptoms	Only 3+ months, duration 2+ months
Musculoskeletal pain	119 (10.5)	24 (2.1)
Cardiovascular	54 (4.8)	6 (0.5)
Respiratory	490 (43.4)	83 (7.4)
Neurological and cognitive dysfunction	313 (27.7)	51 (4.5)
Dermatological	76 (6.7)	8 (0.7)
Gastrointestinal	250 (22.1)	37 (3.2)
Sensory	23 (2.0)	2 (0.2)
Sleep	212 (18.8)	30 (2.7)
Fatigue	244 (21.6)	40 (3.5)
Poor appetite	174 (15.4)	22 (2.0)
*Any symptoms (at least 1)*	*774 (68.6)*	*183 (16.2)*

**Table 3** details the frequency of long COVID symptoms according to gender and age. Most symptoms showed no significant difference between males and females except for cardiovascular and neurological & cognitive dysfunction which were more prevalent in females (p=0.012 and 0.044, respectively). Age-related differences were statistically significant for cardiovascular (p=0.001), neurological & cognitive dysfunction (p<0.001), and fatigue symptoms (p=0.008), which increased with age, while respiratory symptoms (p=0.046) decreased with age​​.

**Table 3:** Frequency of long COVID symptoms in categories, by sex and age classes, n (%).

Symptom	Male	Female	p-value*	0-1y	2-5y	6-11y	12-17y	p-value*
Musculoskeletal pain (n=24)	14 (58.3)	10 (41.7)	0.544	0 (-)	1 (4.2)	17 (70.8)	6 (25.0)	0.063
Cardiovascular (n=6)	0 (-)	6 (100.0)	0.012	0 (-)	0 (-)	1 (16.7)	5 (83.3)	0.001
Respiratory (n=83)	43 (51.8)	40 (48.2)	1.000	17 (20.5)	17 (20.5)	36 (43.4)	13 (15.7)	0.046
Neurological and cognitive dysfunction (n=51)	19 (37.3)	32 (62.8)	0.044	0 (-)	4 (7.8)	29 (56.9)	18 (35.3)	<0.001
Dermatological (n=8)	6 (75.0)	2 (25.0)	0.290	1 (12.5)	0 (-)	5 (62.5)	2 (25.0)	0.642
Gastrointestinal (n=37)	20 (54.1)	17 (46.0)	0.868	3 (8.1)	8 (21.6)	19 (51.4)	7 (18.9)	0.757
Sensory (n=2)	1 (50.0)	1 (50.0)	0.731	0 (-)	0 (-)	0 (-)	2 (100.0)	0.027
Sleep (n=30)	14 (46.7)	16 (53.3)	0.584	2 (6.7)	4 (13.3)	14 (46.7)	10 (33.3)	0.150
Fatigue (n=40)	19 (47.5)	21 (52.5)	0.630	2 (5.0)	2 (5.0)	22 (55.0)	14 (35.0)	0.008
Poor appetite (n=22)	13 (59.1)	9 (40.2)	0.526	3 (13.6)	4 (18.2)	10 (45.5)	5 (22.7)	0.828
*Any symptoms (at least 1) (n=183)*	91 (49.7)	92 (50.3)	0.572	21 (11.5)	28 (15.3)	94 (51.4)	40 (21.9)	0.500

y, years old. *Fisher Exact test or Pearson chi-square test.

### 3.3 Time-to-event analysis

**Figure 1:**
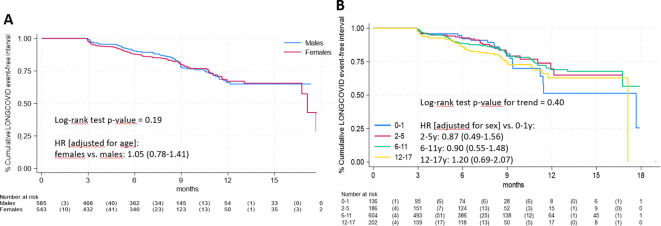
Time-to-event analysis of long COVID symptoms according to gender **(A)** and age group **(B)**.

Median follow-up time was 8.2 months (interquartile range: 5.1-9.3). **Figure 1** presents the time-to-event analysis results. There was no significant difference between males and females (panel A, log-rank test p-value = 0.19) and no significant trend across age groups (panel B, log-rank test p-value = 0.40).

**Figure 2:**
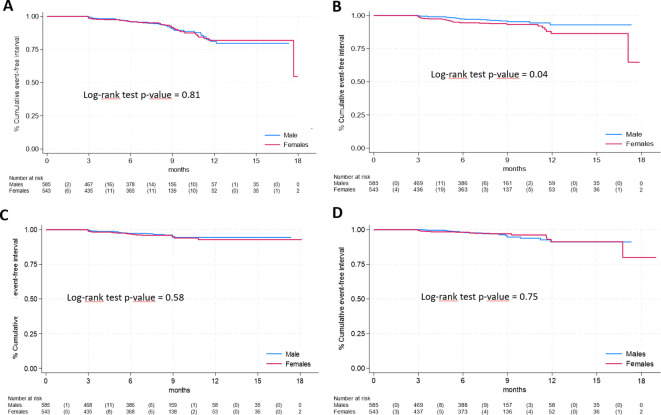
Time-to-event analysis of specific long COVID symptoms according to gender **(A)**, respiratory symptoms; **(B)**, neurological & cognitive dysfunction; **(C)**, fatigue; **(D)**, gastrointestinal symptoms).

Regarding specific long COVID symptoms by gender, Kaplan-Meier curves for the 4 most frequent symptoms are shown in **Figure 2**, ordered by decreasing frequency of symptoms. Only neurological & cognitive dysfunction (panel B) were significantly more frequent in females (log-rank test p-values = 0.04). No statistically significant differences were found for respiratory symptoms (panel A, log-rank test p-value = 0.81), fatigue (panel C, p=0.58) and gastrointestinal symptoms (panel D, p=0.75). Among less frequent symptoms (**Supplementary Figure 1**), significant differences were observed only for cardiovascular symptoms (panel E, log-rank test p-value = 0.01).

**Figure 3:**
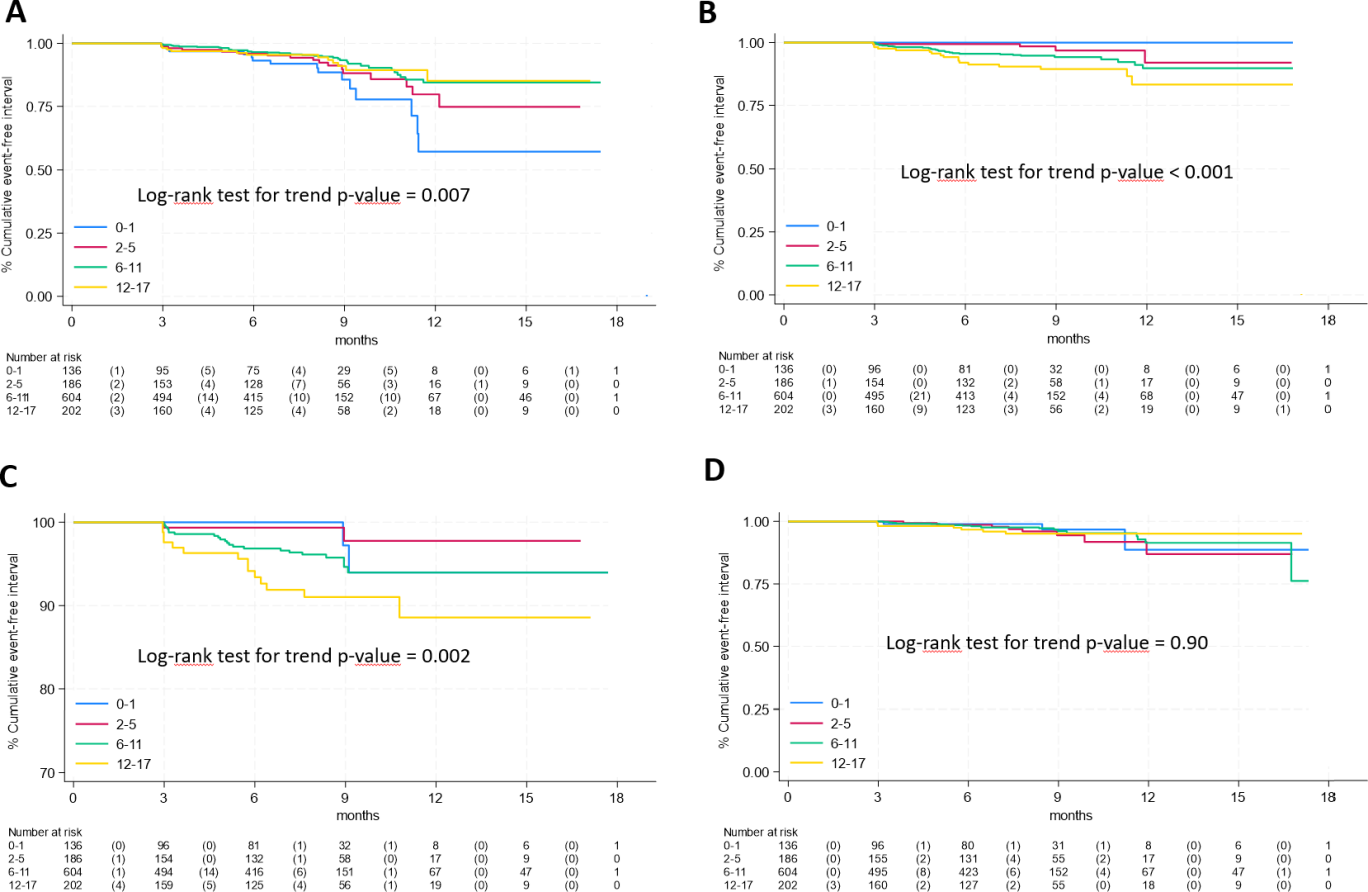
Time-to-event analysis of specific long COVID symptoms according to age group **(A)**, respiratory symptoms; **(B)**, neurological & cognitive dysfunction; **(C)**, fatigue; **(D)**, gastrointestinal symptoms).

Regarding age (**Figure 3**), respiratory symptoms showed a decreased risk with age (panel A, log rank p-value for trend = 0.007) while an overall increased risk with age was observed for neurological & cognitive dysfunction and fatigue (panel B and C respectively, p<0.005 for both), while and no differences among age classes were found for gastrointestinal symptoms (panel D, p=0.90).

In **Supplementary Figure 2** are shown Kaplan Meier curves by age classes for the remaining less frequent symptoms. Older subjects showed higher risks for musculoskeletal pain (panel B, increased risk in age groups 6-17 years, p-value = 0.02), cardiovascular symptoms (panel E, significantly higher in the 12-17 years age group, p-value = 0.003) and, with borderline significance, for sleep disorders (panel A, p=0.08) and sensory symptoms (panel F, p=0.05). No differences among age classes were observed for poor appetite (panel C, p = 0.82) and dermatological symptoms​​ (panel D, p = 0.63).

## 4 Discussion

The study highlights the occurrence of long-term symptoms in children following COVID-19 infection, with significant variations observed based on age and sex for certain symptoms. Notably, neurological and cognitive symptoms, fatigue, and cardiovascular symptoms were higher in older children and females, underscoring the need for targeted follow-up and care in these groups.

The frequency and characteristics of pediatric LC were evaluated using the definition specifically prepared for children by Seylanova et al. (22). The occurrence

was 16.2%, and the type and frequency of clinical manifestations did not differ significantly. These findings confirm that the criteria prepared by Seylanova et al. are well-aligned with WHO criteria and highlight that pediatric LC shares clinical similarities with adult LC. This alignment supports the idea that, despite differences in the initial presentation of COVID-19 between children and adults, the long-term effects can be comparable.

Interestingly, while most studies on adult LC report valueshigher than 16.2%, our results suggest that LC may be less frequent in children. This discrepancy might be attributed to the generally milder course of COVID-19 in pediatric populations, which has been identified as a risk factor for the development of LC (23). Given that COVID-19 is typically less severe in children, it is not surprising that they develop LC less frequently than adults. Our study also confirms that the signs and symptoms of pediatric LC can persist for 3-6 months or even up to 12 months, mirroring patterns observed in adults. This finding emphasizes the need for long-term monitoring and support for children recovering from COVID-19.

The occurrence of LC in our study is within the range reported in previous studies, albeit among the lower end. For example, Lopez-Leon et al. reported a global LC frequency of 25.24% in a meta-analysis of 21 retrospective studies involving 80,071 children (6). Similarly, Zheng et al. found an occurrence of 23.36% in their analysis of 40 retrospective studies with 12,424 children (24). The variation in rates across studies likely reflects differences in study design, recruitment sites, pre-existing conditions, severity of acute COVID-19, follow-up duration, and criteria used to diagnose SARS-CoV-2 infection and define LC.

Our study was conducted in the first trimester of 2022, when over 90% of reported COVID-19 cases in Italy were due to the Omicron variant (25). The lower virulence of the Omicron variant in children, along with the mild to moderate severity of COVID-19 in our study cohort (only 11% required hospitalization), might partly explain the lower occurrence of LC observed (26). This is supported by findings from Morello et al., which indicated a higher likelihood of pediatric LC with pre-Omicron variants (27).

The impact of COVID-19 vaccines on pediatric LC development remains unresolved in our study. Despite evidence from adult studies suggesting that vaccines can reduce the risk of LC (28), the number of vaccinated children in our study was too low to draw definitive conclusions. Given that vaccines have been found effective in reducing COVID-19 incidence and severity in children (29–31), it is plausible that they could also reduce the frequency of LC, a hypothesis that warrants further investigation.

The clinical manifestations of pediatric LC observed in our study are consistent with those reported in other studies, including respiratory symptoms, neuropsychiatric manifestations such as fatigue and sleep disorders, and gastrointestinal symptoms (3, 7, 32, 33). Some physical symptoms, particularly respiratory tract symptoms like rhinitis, might also be attributed to concurrent respiratory viral infections, which are common during the winter season when our study was conducted. The broad spectrum of LC symptoms can be explained by the various pathophysiological mechanisms of SARS-CoV-2, which can cause organ and tissue damage through immune dysregulation, mitochondrial fragmentation, and chronic inflammation (6, 32, 34, 35). Additionally, the mental health and behavioral issues frequently observed in children with LC may be influenced by the psychological and social impacts of the pandemic, including fear of the virus and the effects of mitigation measures (3, 36, 37). The anxious feeling of fear of COVID-19 for themselves, family members, relatives, and friends may have played a significant role in this regard (38). These factors have been found associated with the development of chronic stress, a condition that can induce significant modifications of central nervous system structure and function (39). Memory, cognition, learning and the activity of several body organ and systems can be significantly impaired. Moreover, autonomic dysfunction, i.e. the dysfunction of the sympathetic and/or parasympathetic autonomic nervous system can develop. In most LC cases clinical manifestation such as mood, fatigue, sleep disorders, orthostatic intolerance, decreased concentration, confusion, memory loss, exercise intolerance, body temperature dysregulation, heart rate variability and palpitations, and dysphagia are directly ascribed to the chronic stress and can be considered totally independent from the SARS-CoV-2 infection (40). A relevant part of children and adolescents enrolled in our study suffered from one of more of these clinical manifestations with significant reduction of daily activities and quality of life, highlighting the importance of mental health monitoring in children with COVID-19.

Our study has limitations. Firstly, the lack of a control group of uninfected children monitored during the same period did not enable us to distinguish symptoms directly related to SARS-CoV-2 from those associated with the broader impacts of the pandemic. However, the standardized follow-up data collection protocol followed in this study was developed by ISARIC for the rapid, coordinated clinical investigation of severe illness caused by emerging pathogens, and did not include a control group [https://isaric.org/research/covid-19-clinical-research-resources/]. Secondly, data collection through questionnaires can introduce biases, although we minimized this by using validated protocols developed for evaluating LC in adults. Furthermore, the large sample size and prospective design strengthen the reliability of our findings, allowing us to capture symptom persistence over time with a systematic follow-up approach. Despite the well-known limitations of self-reported data, we should also point out that the use of PROs can represent a strength of studies on LC, which is in many ways a subjective experience that can be underestimated by clinicians. Finally, another potential limitation is the inclusion period between January and March, which coincides with the peak season for respiratory infections. This timing could have influenced the overall frequency of respiratory symptoms, particularly if all symptoms were considered rather than only those persisting for more than two months. However, our analysis specifically focused on symptoms that lasted consecutively for more than two months and occur within three months after the initial SARS-CoV-2 infection not explained by another diagnosis, reducing the likelihood of seasonal confounders affecting the assessment of long COVID.

## 5 Conclusions

Our findings contribute to the growing understanding of pediatric LC, and suggest the need to monitor children with persisting problems. Electronic monitoring with standardized questionnaires and alerts to clinicians in case of symptom worsening during pandemic emergencies should be considered.

Further research is needed to elucidate the long-term impacts of COVID-19 in pediatric populations, the potential protective effects of vaccination, and the impact of different SARS-CoV-2 variants on the prevalence and nature of pediatric LC.

## Data Availability

The raw data supporting the conclusions of this article will be made available by the authors, without undue reservation.

## Long-Covid-Ped Italian Study Group

Long-Covid-Ped Italian Study Group includes also the following researchers: Lucia Forino (Napoli); Valentina Talarico (Catanzaro); Sara Bagarello (Palermo); Cristina Forest, Cristina Malaventura (Ferrara); Alessandra Pisani, Francesco Carella, Valentina Palladino (Bari); Giulia Molari, Giada Di Pietro (Milano); Shana Montalto, Benedetta Schiappapietra (Genova); Enrica Mancino, Luigi Matera, Greta Di Mattia (Roma); Marica De Pieri, Costanza Di Chiara, Giulia Sturniolo (Padova); Maria Elena Capra, Nicoletta De Paulis (Piacenza).

## Supplementary material

Supplementary Figure 1 Time-to-event analysis of specific long COVID symptoms according to gender **(A)**, sleep; **(B)**, musculoskeletal pain; **(C)**, poor appetite; **(D)**, dermatological; **(E)**, cardiovascular; **(F)**, sensory).

Supplementary Figure 2 Time-to-event analysis of specific long COVID symptoms according to age group **(A)**, sleep; **(B)**, musculoskeletal pain; **(C)**, poor appetite; **(D)**, dermatological; **(E)**, cardiovascular; **(F)**, sensory).
