# Supplementary material for: Long COVID in pediatric age: an observational, prospective, longitudinal, multicenter study in Italy
Source: Front Immunol. 2025 Apr 9;16:1466201. doi: 10.3389/fimmu.2025.1466201 (PMC12015939; doi:10.3389/fimmu.2025.1466201)
Supplement: Supplementary Figure 1 — Time-to-event analysis of specific long COVID symptoms according to gender (A), sleep; (B), musculoskeletal pain; (C), poor appetite; (D), dermatological; (E), cardiovascular; (F), sensory). [file Presentation1.pptx]

## Slide 1
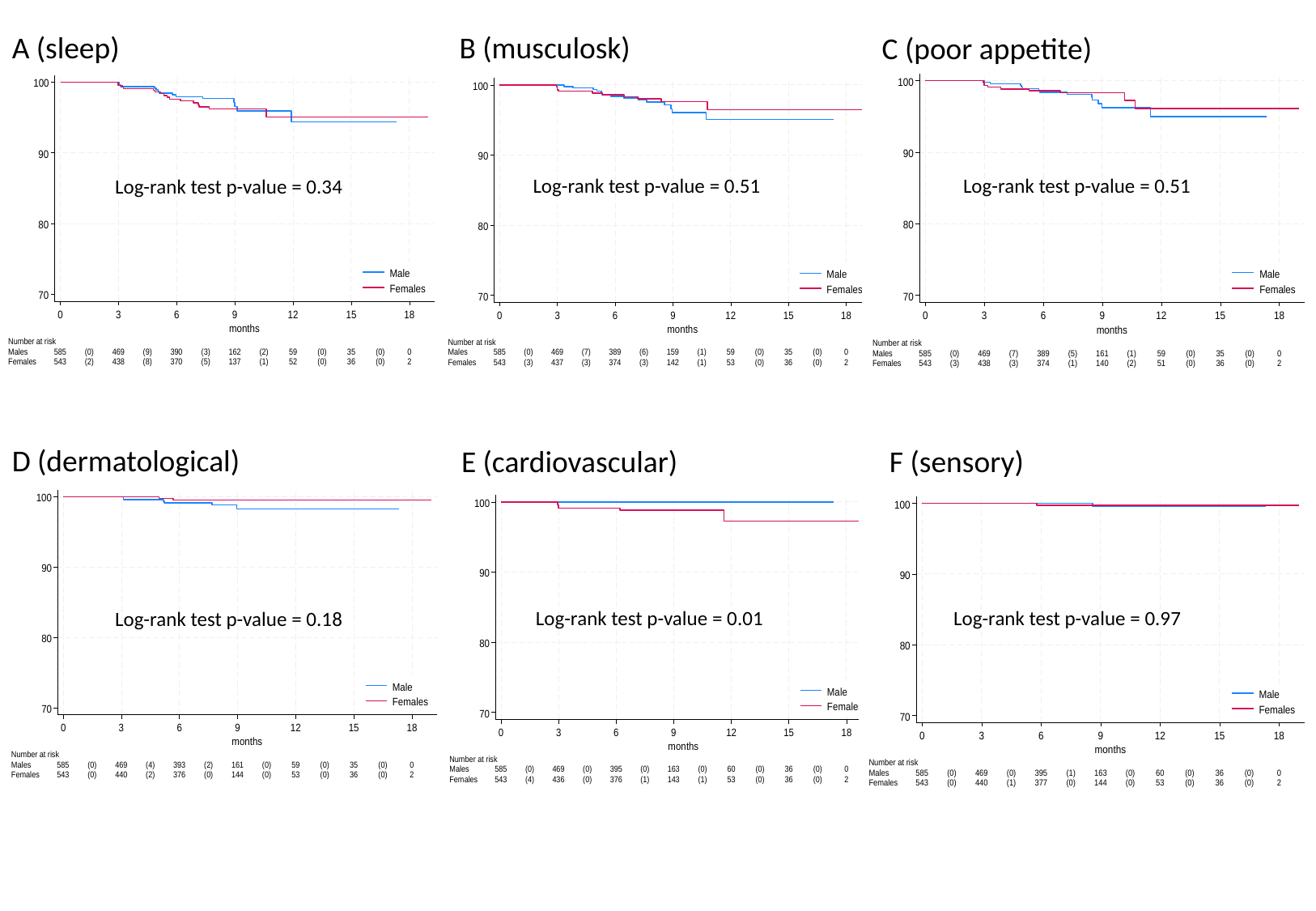

A (sleep)
B (musculosk)
C (poor appetite)
Log-rank test p-value = 0.51
Log-rank test p-value = 0.51
Log-rank test p-value = 0.34
D (dermatological)
E (cardiovascular)
F (sensory)
Log-rank test p-value = 0.01
Log-rank test p-value = 0.97
Log-rank test p-value = 0.18
