# Supplementary material for: Long COVID in pediatric age: an observational, prospective, longitudinal, multicenter study in Italy
Source: Front Immunol. 2025 Apr 9;16:1466201. doi: 10.3389/fimmu.2025.1466201 (PMC12015939; doi:10.3389/fimmu.2025.1466201)
Supplement: Supplementary file 2 [file Table1.docx]

| **Supplementary Table 1.** Categorisation of post-COVID-19 symptoms. | |
| --- | --- |
| **Category** | **Symptoms** |
| Musculoskeletal pain | Persistent muscle pain, Joint pain or swelling |
| Cardiovascular | Palpitations (heartbeat), Variation in heart rate (tachycardia or bradycardia) |
| Respiratory | Difficulty breathing/chest compressions, Chest pain, Persistent cough, Nasal congestion/rhinorrhoea |
| Neurological and cognitive dysfunction | Problems with balance, Tingling sensation, Confusion/loss of concentration, Fainting, Dizziness/ dizziness, Vision problems/blurred vision, Headaches |
| Dermatological | Skin rash |
| Gastrointestinal | Constipation - diarrhoea, Stomach/abdominal pain, Persistent feeling of nausea/Vomiting |
| Sensory | Disturbed smell/loss of smell, Disturbed taste/loss of taste |
| Sleep | Insomnia (difficult to fall asleep, difficult to sleep), Hypersomnia (excessive daytime sleepiness, prolonged sleepiness at night) |
| Fatigue | Fatigue |
| Poor appetite | Poor appetite |
